# Supplementary material for: Measurement protocols, random-variable-valued measurements, and response process error: Estimation and inference when sample data are not deterministic
Source: PLoS One. 2020 Oct 1;15(10):e0239821. doi: 10.1371/journal.pone.0239821 (PMC7529193; doi:10.1371/journal.pone.0239821)
Supplement: S1 Appendix — (PDF) [file pone.0239821.s001.pdf]

S1 Appendix. Proofs of propositions.

Proof of Proposition 2.3. Since ρ is calibrated to Y, we know that

$$\int_{\mathbb{R}} x \; d\mu_{\omega_i}(x) = \mathbb{E}_{(\Omega,\mathscr{F})}(Y \mid \mathscr{O}_{\omega_i})$$

for each i. But $\mathbb{E}(\mathbb{E}(Y \mid \mathscr{O}_{\omega_i})) = \mathbb{E}(Y)$, and so the estimator is unbiased.

Next, recall that we could just as easily write

$$\int_{\mathbb{R}} x \; d\mu_{\omega_i}(x) = \mathbb{E}_{(\mathbb{R},\mathscr{B}(\mathbb{R}))}(X_i)$$

where $X_i \sim \mu_{\omega_i}$. Now, using the fact that our measurements are independent, the definition of calibration, and the generic expression for total variance, we simply calculate

$$\begin{aligned}
\mathrm{Var}(\overline{\rho}(\mathscr{S})) &= \frac{1}{n^2}\sum_{i=1}^{n} \mathrm{Var}_{(\Omega,\mathscr{F})}[\mathbb{E}_{(\mathbb{R},\mathscr{B}(\mathbb{R}))}(X_i)] \\
&= \frac{1}{n^2}\sum_{i=1}^{n} \mathrm{Var}_{(\Omega,\mathscr{F})}[\mathbb{E}_{(\Omega,\mathscr{F})}(Y \mid \mathscr{O}_{\omega_i})] \\
&= \frac{\mathrm{Var}(Y)}{n} - \frac{1}{n^2}\sum_{i=1}^{n} \mathbb{E}[\mathrm{Var}(Y \mid \mathscr{O}_{\omega_i})]. \qquad (10)
\end{aligned}$$

But now since $0 \leq \mathbb{E}[\mathrm{Var}(Y \mid \mathscr{O}_{\omega})] \leq \mathrm{Var}(Y)$, we must have the expression in (10) bounded by 0 and $\mathrm{Var}(Y)/n$; i.e. the traditional standard error of the mean generated by a sample of fixed measurements. Consistency follows. □

Proof of Proposition 3.1. Since ν is the convolution of the independent measures $\mu_1, \ldots, \mu_n$, we have

$$n\overline{\rho}(\mathscr{S}) = \sum_{i=1}^{n} \int_{\mathbb{R}} z \; d\mu_i(z) = \mathbb{E}_{\nu}(W).$$

At the same time, by the definition of a calibrated measurement protocol, we have

$$n\mathbb{E}(\overline{\rho}(\mathscr{S})) = n\mathbb{E}(Y) = n\theta_0.$$

Therefore, by (8), we have

$$\mathbb{E}(\widehat{\theta}) = \frac{\alpha + \mathbb{E}[\mathbb{E}_\nu(W)]}{\alpha+\beta+n} = \frac{\alpha + n\theta_0}{\alpha+\beta+n},$$

which approaches θ_0 as $n \to \infty$. □

Proof of Proposition 3.2. Since trivial RVVMs free of measurement error are automatically calibrated (see Section 2.2), part (i) follows immediately from Proposition 3.1. Note too that this implies that the bias of $\widehat{\theta}$ is the same as what would be generated by the classical Bayes' estimator derived from the measurement protocol that yields complete information RVVMs free of measurement error. That is, calibrated nontrivial RVVMs do not induce any additional bias that would not have already been present in the error-free sample data $y_1, \ldots, y_n$.

If instead we only know that $m_2 = o(m_1)$, then we may first simplify the expression for the Bayes' estimator in (8). Using the customary notation, we have

$$\mathbb{E}(\theta \mid \rho(\mathscr{S})) = \frac{\alpha + \sum_{i=1}^{m_1} y_i}{\alpha+\beta+m_1+m_2} + \frac{\mathbb{E}_\nu(W)}{\alpha+\beta+m_1+m_2},$$

where we have redefined $\nu = \mu_{n-m_2+1} * \cdots * \mu_n$, with $W \sim \nu$, by separating the first m_1 point-mass RVVMs from the nontrivial response process measure ν. The expectation of the first term is $(\alpha + m_1\theta_0)(\alpha+\beta+m_1+m_2)^{-1}$ which approaches θ_0 as $m_1 + m_2 \to \infty$, while the second term is bounded by $m_2(\alpha+\beta+m_1+m_2)^{-1}$. Since $m_2 = o(m_1)$, this term goes to zero as $m_1 + m_2 \to \infty$. Thus, $\mathbb{E}(\widehat{\theta}) \to \theta_0$, proving (ii).

To prove the final piece of the proposition, we use our expression for the posterior variance (9) and rewrite in terms of the redefined ν:

$$\begin{aligned}\mathrm{Var}(\theta \mid \rho(\mathscr{S})) = {} & \frac{\alpha(\beta+n) + (\beta+n-\alpha)(\sum_{i=1}^{m_1} y_i + \mathbb{E}_\nu(W)) + (\sum_{i=1}^{m_1} y_i + \mathbb{E}_\nu(W))^2}{(\alpha+\beta+n)^2(\alpha+\beta+n+1)} \\ & + \frac{\mathrm{Var}_\nu(\sum_{i=1}^{m_1} y_i + W)}{(\alpha+\beta+n)(\alpha+\beta+n+1)}. \end{aligned} \tag{11}$$

Using again the fact that $\mathbb{E}_\nu(W) \le m_2$, the first term of this expression is asymptotically equivalent to $(m_1+m_2)^{-1}$. Thus, this term goes to zero as $(m_1+m_2) \to \infty$ regardless of the growth rate of m_2. Now, $\mathrm{Var}(\theta \mid \rho(\mathscr{S}), W)$ is always bounded by a constant function, which is integrable over ν, thus by the reverse Fatou's Lemma, $\mathbb{E}_\nu[\mathrm{Var}(\theta \mid \rho(\mathscr{S}), W)] \to 0$.

To deal with the second term in equation (11), we first simplify to the bound

$$\le \mathrm{Var}_\nu\left(\frac{\sum_{i=1}^{m_1} y_i + W}{\alpha+\beta+n}\right),$$

and then apply Popoviciu's Inequality:

$$\le \frac{1}{4}\left(\frac{\alpha + \sum_{i=1}^{m_1} y_i + m_2}{\alpha+\beta+m_1+m_2} - \frac{\alpha + \sum_{i=1}^{m_1} y_i}{\alpha+\beta+m_1+m_2}\right)^2.$$

Thus,

$$\mathrm{Var}_\nu[\mathbb{E}(\theta \mid \rho(\mathscr{S}), W)] \le \frac{m_2^2}{4(\alpha+\beta+m_1+m_2)^2}.$$

This bound is asymptotically equivalent to $m_2^2(m_1+m_2)^{-2}$ which goes to zero if $m_2 = o(m_1)$. Therefore, $\mathrm{Var}(\theta \mid \rho(\mathscr{S})) \to 0$ if $m_2 = o(m_1)$, proving the last part of the proposition. □
