# Supplementary material for: Measurement protocols, random-variable-valued measurements, and response process error: Estimation and inference when sample data are not deterministic
Source: PLoS One. 2020 Oct 1;15(10):e0239821. doi: 10.1371/journal.pone.0239821 (PMC7529193; doi:10.1371/journal.pone.0239821)
Supplement: S2 Appendix — (PDF) [file pone.0239821.s002.pdf]

S2 Appendix. R code for worked examples.

$Y \sim Ber(p)$, the sex distribution of a species at a location.
$Y|\text{adult} \sim Ber(.5)$.
$Y|\text{juvenile} \sim Ber(.75)$.
Juveniles are difficult to sex. Age is always definitive.
Adults outnumber juveniles by about 4 to 1.
Total sample size is 50 birds: 12 juveniles and 38 adults.

```
### Create 'age' and 'sex' data; define mean
        and sd functions for betas ###
age <- c(rep(1,38),rep(0,12))
sex <- c(rep(1,19),rep(0,19),rep(1,9),rep(0,3))
dat <- data.frame(age,sex)
meanb <- function(a,b,x,n){(a+x)/(a+b+n)}
sdb <- function(a,b,x,n){sqrt(((a+x)*(b+n-x))/
        ((a+b+n+1)*(a+b+n)^2))}
###
```

All adults are definitively sexed (fixed measurements).
Of 12 juveniles, only 4 are definitively sexed, all 4 females.
The remaining 8 juveniles generate Bernolli-valued measurements:
Bernoulli-valued measurements generated to be calibrated on the sample (ideal scenario):

$\rho(\omega_{43}) \sim Ber(0.9),\ \rho(\omega_{44}) \sim Ber(0.9),\ \rho(\omega_{45}) \sim Ber(0.8),\ \rho(\omega_{46}) \sim Ber(0.8),$
$\rho(\omega_{47}) \sim Ber(0.7),\ \rho(\omega_{48}) \sim Ber(0.4),\ \rho(\omega_{49}) \sim Ber(0.3),\ \rho(\omega_{50}) \sim Ber(0.2).$

```
### Full fixed data (unobserved) estimates ###
meanb(15,15,sum(dat$sex),50)
sdb(15,15,sum(dat$sex),50)
###
```

Create 'sex.obs' variable with missing data in place of nontrivial RVVMs (measurement protocol 2). Then estimate sample proportions:

```
###
dat$sex.obs <- c(dat$sex[1:42],rep(NA,8))
dat.red <- dat[,c(1,4)]
###
```

Estimates under measurement protocol 2

```
###
meanb(15,15,sum(na.omit(dat$sex.obs)),42)
sdb(15,15,sum(na.omit(dat$sex.obs)),42)
###
```

```
###
### Generate all sample instantiations of the RVVMs on the ###
### auxiliary measurable space ###
dat$rvvm <- c(sex[1:42],0.9,0.9,0.8,0.8,0.7,0.4,0.3,0.2)
aux.sample.space <- expand.grid(replicate(8, 0:1,
        simplify = FALSE))
rvvm.sample <- matrix(nrow=50,ncol=256)
for(i in 1:256){ rvvm.sample[,i] <- c(sex[1:42],
        as.numeric(aux.sample.space[i,])) }
```

```
### RVVM-based estimates ###
varb <- function(a,b,x,n){((a+x)*(b+n-x))/((a+b+n+1)*(a+b+n)^2)}
prob <- c(rep(0,256))
for(i in 1:256){
prob[i] <- prod(dat$rvvm^(rvvm.sample[,i])
        *(1-dat$rvvm)^(1-rvvm.sample[,i]))
}
count <- matrix(rep(0,256),ncol=256)
variance <- matrix(rep(0,256),ncol=256)
exp <- matrix(rep(0,256),ncol=256)
for(m in 1:256){
count[m] <- sum(rvvm.sample[,m])
exp[m] <- meanb(15,15,count[m],50)
variance[m] <- varb(15,15,count[m],50)
}
mean <- sum(exp*prob)
sd <- sqrt(sum(variance*prob) + sum(prob*(exp-sum(exp*prob))^2))
###
```

Fit *sex* $\sim$ *age* logistic models.

```
### Load libraries ###
library(rstanarm)
library(mice)

### Fit with full true data (unobserved); measurement
        protocol 1 ###
set.seed(3323)
post.true <- stan_glm(sex~age, family=binomial, data=dat)
coef(post.true)
sqrt(diag(post.true$covmat))
exp(sum(coef(post.true)))
exp(coef(post.true)[1])

### Fit with response error-free data only; measurement
        protocol 2 ###
set.seed(3323)
post.naive <- stan_glm(sex[1:42]~age[1:42], family=binomial,
        data=dat)
coef(post.naive)
sqrt(diag(post.naive$covmat))
exp(sum(coef(post.naive)))
exp(coef(post.naive)[1])

### Fit with response error-free data only and treating
        response error-prone data as ``missing'' (imputation);
        measurement protocol 2 ######
set.seed(3323)
imputed <- mice::mice(dat.red, m=5, method="pmm", maxit=10,
        seed=12345)
mod.imp <- with(imputed, stan_glm(sex.obs ~ age,
        family=binomial))
pool(mod.imp)
```

```
### RVVM-based fits , measurement protocol 3 ###
set.seed(3323)
prob <- c(rep(0,256))
for(i in 1:256){
prob[i] <- prod(dat$rvvm^(rvvm.sample[,i])
        *(1-dat$rvvm)^(1-rvvm.sample[,i]))
}
coefficient <- matrix(rep(0,256*2),ncol=2)
variance <- matrix(rep(0,256*2),ncol=2)
for(m in 1:256){
post <- stan_glm(rvvm.sample[,m]~age,family=binomial,data=dat)
print(m)
coefficient[m,] <- coef(post)
variance[m,] <- diag(post$covmat)
}
post.exp <- c(rep(0,2))
post.var <- c(rep(0,2))
for(i in 1:2){
        post.exp[i] <- sum(coefficient[,i]*prob)
        post.var[i] <- sum(variance[,i]*prob)
                + sum((coefficient[,i]-post.exp[i])^2*prob)
}
post.exp
sqrt(post.var)
exp(sum(post.exp))
exp(post.exp[1])
###
```

Create the ‘weight’ and ‘wing chord’ variables:

```
###
set.seed(2523)
weight <- c(rep(0,50))
for(i in 1:42){
if(dat$sex[i]==1 && dat$age[i]==0)
        {weight[i] <- rnorm(1,30,5)}
else if(dat$sex[i]==0 && dat$age[i]==0)
        {weight[i] <- rnorm(1,40,5)}
else if(dat$sex[i]==1 && dat$age[i]==1)
        {weight[i] <- rnorm(1,50,5)}
else if(dat$sex[i]==0 && dat$age[i]==1)
        {weight[i] <- rnorm(1,60,5)}
}
for(i in 43:50){
if(dat$sex[i]==1){weight[i] <- rnorm(1,20,5)}
else if(dat$sex[i]==0){weight[i] <- rnorm(1,30,5)}
}
dat$weight <- weight
###
###
wc <- c(rep(50))
for(i in 1:50){
if(dat$sex[i]==0 && dat$age[i]==0){wc[i] <- rnorm(1,8,1)}
else {wc[i] <- rnorm(1,11,1)}
```

```
}
dat$wc <- wc
###
```

Fit $sex \sim age * weight$ logistic models.

```
### Fit with full true data (unobserved), measurement
        protocol 1 ###
set.seed(3323)
post.true <- stan_glm(sex~age*weight, family=binomial,
        data=dat)
coef(post.true)
sqrt(diag(post.true$covmat))
exp(coef(post.true)[1] + coef(post.true)[3]*25)
exp(coef(post.true)[1] + coef(post.true)[3]*30)
exp(coef(post.true)[1] + coef(post.true)[2]
        + coef(post.true)[3]*50 + coef(post.true)[4]*50)
exp(coef(post.true)[1] + coef(post.true)[2]
        + coef(post.true)[3]*60 + coef(post.true)[4]*60)

### Observed fixed data with imputations, measurement
        protocol 2 ###
sex.obs <- dat$sex.obs
dat.red <- data.frame(age,sex.obs,weight)
imputed <- mice::mice(dat.red, m=5, method="pmm", maxit=10,
        seed=12345)
mod.imp <- with(imputed, stan_glm(sex.obs ~ age*weight,
         family=binomial))
p <- pool(mod.imp)$pooled$estimate
p
sqrt(pool(mod.imp)$pooled$ubar)
exp(p[1] + p[3]*25)
exp(p[1] + p[3]*30)
exp(p[1] + p[2] + p[3]*50
        + p[4]*50)
exp(p[1] + p[2] + p[3]*60
        + p[4]*60)

### Observed fixed data with NO imputations,
        measurement protocol 2 ###
dat.red2 <- dat.red[1:42,]
post.true <- stan_glm(dat.red2$sex~dat.red2$age*dat.red2$weight,
        family=binomial)
coef(post.true)
sqrt(diag(post.true$covmat))
exp(coef(post.true)[1] + coef(post.true)[3]*25)
exp(coef(post.true)[1] + coef(post.true)[3]*30)
exp(coef(post.true)[1] + coef(post.true)[2]
        + coef(post.true)[3]*50 + coef(post.true)[4]*50)
exp(coef(post.true)[1] + coef(post.true)[2]
        + coef(post.true)[3]*60 + coef(post.true)[4]*60)

### RVVM-based fits, measurement protocol 3 ###
set.seed(3323)
```

```
prob <- c(rep(0,256))
for(i in 1:256){
prob[i] <- prod(dat$rvvm^(rvvm.sample[,i])
        *(1-dat$rvvm)^(1-rvvm.sample[,i]))
}
coefficient <- matrix(rep(0,256*4),ncol=4)
variance <- matrix(rep(0,256*4),ncol=4)
for(m in 1:256){
post <- stan_glm(rvvm.sample[,m]~age*weight,family=binomial,
        data=dat)
print(m)
coefficient[m,] <- coef(post)
variance[m,] <- diag(post$covmat)
}
post.exp <- c(rep(0,4))
post.var <- c(rep(0,4))
for(i in 1:4){
        post.exp[i] <- sum(coefficient[,i]*prob)
        post.var[i] <- sum(variance[,i]*prob)
                + sum((coefficient[,i]-post.exp[i])^2*prob)
}
post.exp
sqrt(post.var)
exp(post.exp[1] + post.exp[3]*25)
exp(post.exp[1] + post.exp[3]*30)
exp(post.exp[1] + post.exp[2] + post.exp[3]*50 + post.exp[4]*50)
exp(post.exp[1] + post.exp[2] + post.exp[3]*60 + post.exp[4]*60)
###
```

Fit $sex \sim age * wc$ logistic models.

```
### Fit with full true data (unobserved), measurement
        protocol 1 ###
set.seed(323)
post.true <- stan_glm(sex~age*wc, family=binomial, data=dat)
coef(post.true)
sqrt(diag(post.true$covmat))
exp(coef(post.true)[1] + coef(post.true)[3]*8)
exp(coef(post.true)[1] + coef(post.true)[3]*11)
exp(coef(post.true)[1] + coef(post.true)[2]
        + coef(post.true)[3]*11 + coef(post.true)[4]*11)

### Observed fixed data with imputations, measurement
        protocol 2 ###
dat.red <- data.frame(age,sex.obs,wc)
imputed <- mice::mice(dat.red, m=5, method="pmm",
        maxit=10, seed=12345)
mod.imp <- with(imputed, stan_glm(sex.obs ~ age*wc,
        family=binomial))
p <- pool(mod.imp)$pooled$estimate
p
sqrt(pool(mod.imp)$pooled$ubar)
exp(p[1] + p[3]*8)
exp(p[1] + p[3]*11)
```

```
exp(p[1] + p[2] + p[3]*11
        + p[4]*11)

### Observed fixed data with NO imputations, measurement
        protocol 2 ###
dat.red2 <- dat.red[1:42,]
post.true <- stan_glm(dat.red2$sex~dat.red2$age*dat.red2$wc,
        family=binomial)
coef(post.true)
sqrt(diag(post.true$covmat))
exp(coef(post.true)[1] + coef(post.true)[3]*8)
exp(coef(post.true)[1] + coef(post.true)[3]*11)
exp(coef(post.true)[1] + coef(post.true)[2]
        + coef(post.true)[3]*11 + coef(post.true)[4]*11)


### RVVM-based fits, measurement protocol 3 ###
set.seed(3323)
prob <- c(rep(0,256))
for(i in 1:256){
prob[i] <- prod(dat$rvvm^(rvvm.sample[,i])
        *(1-dat$rvvm)^(1-rvvm.sample[,i]))
}
coefficient <- matrix(rep(0,256*4),ncol=4)
variance <- matrix(rep(0,256*4),ncol=4)
for(m in 1:256){
post <- stan_glm(rvvm.sample[,m]~age*wc,family=binomial,
        data=dat)
print(m)
coefficient[m,] <- coef(post)
variance[m,] <- diag(post$covmat)
}
post.exp <- c(rep(0,4))
post.var <- c(rep(0,4))
for(i in 1:4){
        post.exp[i] <- sum(coefficient[,i]*prob)
        post.var[i] <- sum(variance[,i]*prob)
                + sum((coefficient[,i]-post.exp[i])^2*prob)
}
post.exp
sqrt(post.var)
exp(post.exp[1] + post.exp[3]*8)
exp(post.exp[1] + post.exp[3]*11)
exp(post.exp[1] + post.exp[2] + post.exp[3]*11 + post.exp[4]*11)
###
```

Fit $wc \sim sex * age$ Gaussian models.

```
### Fit with full true data (unobserved), measurement
        protocol 1 ###
set.seed(3323)
post.true <- stan_glm(wc~sex*age, family=gaussian,
        data=dat)
coef(post.true)
```

```
sqrt(diag(post.true$covmat))
coef(post.true)[1] + coef(post.true)[2]
coef(post.true)[1]
coef(post.true)[1] + coef(post.true)[2] + coef(post.true)[3]
        + coef(post.true)[4]
coef(post.true)[1] + coef(post.true)[3]

### Observed fixed data with imputations, measurement
        protocol 2 ###
dat.red <- data.frame(age,sex.obs,wc)
imputed <- mice::mice(dat.red, m=5, method="pmm",
        maxit=10, seed=12345)
mod.imp <- with(imputed, stan_glm(wc ~ sex.obs*age,
        family=gaussian))
p <- pool(mod.imp)$pooled$estimate
p
sqrt(pool(mod.imp)$pooled$ubar)
p[1] + p[2]
p[1]
p[1] + p[2] + p[3] + p[4]
p[1] + p[3]

### Observed fixed data with NO imputations, measurement
        protocol 2 ###
dat.red2 <- dat.red[1:42,]
post.true <- stan_glm(dat.red2$wc ~
         dat.red2$sex.obs*dat.red2$age, family=gaussian)
coef(post.true)
sqrt(diag(post.true$covmat))
coef(post.true)[1] + coef(post.true)[2]
coef(post.true)[1]
coef(post.true)[1] + coef(post.true)[2]
        + coef(post.true)[3] + coef(post.true)[4]
coef(post.true)[1] + coef(post.true)[3]

### RVVM-based fits, measurement protocol 3 ###
set.seed(3323)
prob <- c(rep(0,256))
for(i in 1:256){
prob[i] <- prod(dat$rvvm^(rvvm.sample[,i])
        *(1-dat$rvvm)^(1-rvvm.sample[,i]))
}
coefficient <- matrix(rep(0,256*4),ncol=4)
variance <- matrix(rep(0,256*4),ncol=4)
for(m in 1:256){
post <- stan_glm(wc ~ rvvm.sample[,m]*age,family=gaussian,
        data=dat)
print(m)
coefficient[m,] <- coef(post)
variance[m,] <- diag(post$covmat)
}
post.exp <- c(rep(0,4))
```

```
post.var <- c(rep(0,4))
for(i in 1:4){
        post.exp[i] <- sum(coefficient[,i]*prob)
        post.var[i] <- sum(variance[,i]*prob)
                + sum((coefficient[,i]-post.exp[i])^2*prob)
}
post.exp
sqrt(post.var)
post.exp[1] + post.exp[2]
post.exp[1]
post.exp[1] + post.exp[2] + post.exp[3] + post.exp[4]
post.exp[1] + post.exp[3]
###
```
