# Supplementary material for: Measurement protocols, random-variable-valued measurements, and response process error: Estimation and inference when sample data are not deterministic
Source: PLoS One. 2020 Oct 1;15(10):e0239821. doi: 10.1371/journal.pone.0239821 (PMC7529193; doi:10.1371/journal.pone.0239821)
Supplement: Attachment — Submitted filename: CoverLetter_PlosOne_ResponseToReviewers.pdf [file pone.0239821.s003.pdf]

Response to Reviewers:

Several large structural changes have been made, per recommendations of the editor and those of the reviewers:

1. The Introduction has been rewritten and slighlty condensed. I have rewritten and expanded slightly on the psychometrics applications of response process error, eliminated the field ecology example (as discussion of this already appeared in Section 4), and clarified the connection between the RVVM approach and the related fuzzy numbers/statistics/information approaches. I have also given the reader more guidance as to the content of each subsequent section of the paper in the final paragraph. I erred on the side of verbosity right now, but if one of the reviewers feels that the Introduction could still benefit from further pruning, I would be happy to oblige.

2. Section 2 is now split into only 2 subsections. Previous Subsections 2.1 through 2.4 have been collapsed into a single subsection, and a few lines have been added to make the exposition flow more naturally. In particular, previous Section 2.2 is now included in Section 2.1 per your recommendation. Section 2.3 has been almost completely excised. You recommended that this section be "pruned and better justified," and this section had bothered me more than any other upon initial submission. After careful review, I was able to restate my point in a single concluding paragraph to the new Section 2.1, and also used it to help motivate the content of Section 3. The previous subsection 2.4 on the information theoretic interpretation of RVVMs has been subsumed in the new Section 2.1, with extra motivation and connections to Section 3 added into the exposition (see the "track changes" document for specifics).

3. The new Section 2.2 is a slight expansion of the previous Section 2.5 on calibrated RVVMs. I have added several paragraphs to emphasize why this idea is so important, and also identified where this content is used in both the theoretical ideas of Bayesian estimation in Section 3, and the applied examples in Section 4. I have also slightly reworded and clarified the meaning of Proposition 2.3. Previously, this seemed to be somewhat of a standalone result, but now I have emphasized how it plays a role in future sections, and also properly identified it as a generalization of the classical Weak Law of Large Numbers applicable to the context of RVVMs. I have also slightly changed the mathematical notation I used in the presentation of these calibration ideas to a format that I feel is more transparent.

4. While I have retained the lengthy bird-sexing example of Section 4, I have attempted to further clarify how the RVVM approach can improve understanding of population demographics in field ecology. I have also added several pages to explore a second research scenario from the social sciences where RVVMs naturally

arise. This is an application from psychometrics and clinical psychology: the application of the classical Hamilton Depression Rating Scale to the (partial/tentative) diagnosis of patients thought to be suffering from depression. Connections back to the calibration section 2.2 are also included.

5. All mathematical proofs of propositions have been moved to a separate Appendix (Section 7).

6. Several new references have been added.

Other points of note:

1. I appreciate the editor and Reviewer 2 pointing out that I improperly impugned and dismissed ideas from "fuzzy statistics" in my initial submission. I believe that I have fixed this in the revision. In the Introduction in particular, I clarify that it is really the *common application* of fuzzy numbers – via the special case of triangular numbers that drives, for instance, virtually all of these ideas in the field of conservation biology – that differs from the RVVM approach. I also clarify that while the fuzzy number approach operates by generalizing numbers to certain kinds of functions (which can be construed as certain kinds of measures), it is really the kind of *sample data* that the two approaches consider that distinguishes them. I also indicate that the RVVM approach has this conceptual distinction in common with the fuzzy information approach due to Okuda, Tanaka, and Asai (Okuda *et al.* 1978 and Tanaka *et al.* 1979). Again, their approach is not concerned with formalizing what a measurement process/operator actually entails in an applied scientific context, but their approach does formalize the idea that sample data need not take the form of only fixed real numbers. In this way, it certainly resembles the RVVM approach that I propose, though they do not use the language of a measure-valued mapping to discuss sample data. Personally, I think it would make a fascinating paper to rigorously compare and contrast the two approaches, though I hope that you and the reviewers agree with me that such an undertaking would be tangential to the purposes of the current paper.

2. Per Reviwer 1's request, I have attempted to clarify the point and implications of the information theoretic interpretation of RVVMs in the new Section 2.1, clarifying also that "information" is meant to be interpreted in the classical Shannon sense.

3. I agree with Reviewer 1 that it would be interesting to investigate how hypothesis testing can operate when working with RVVMs. I have purposely avoided bringing this topic up however, both in my original submission and this revision, because I believe that the topic is a large and nontrivial one. Classical hypothesis testing tends to work by assuming that one can derive a workable distribution for the

test statistic in question. This usually works by assuming that the test statistic is constructed from some kind of arithmetic combination of i.i.d. sample draws from the target phenomenon of interest. However, in the RVVM context, the presence of response process error completely destroys the "identical distribution" aspect of the individual sample measurements; indeed, each sample measurement can be a distinct probability measure that merely reflects information from the target phenomenon of interest in some (presumably) meaningful way. The resulting distribution of any test statistic (under the point null, or otherwise) thus becomes a question about the distribution of (usually) some function of the convolution of many non-identical measures. This is certainly a fascinating line of inquiry, but I continue to think that it would be best broached in a separate paper. In fact, this is another advantage of my choice to take a purely Bayesian approach to inference in the current paper: while Bayesian hypothesis testing is certainly a well established idea, there are plenty of practicing Bayesians that would still reject the whole epistemological principle of hypothesis testing within the Bayesian context (full disclosure: I would consider myself one of those Bayesians, as would several people I have worked with). The examples of Section 4.1 are only concerned with point estimation of posterior means and standard deviations. I am purposely silent on any binary decision-making ideas from the tradition of hypothesis testing.

4. I am not sure why Reviewer 2 has indicated in his/her review that my results are not technically sound and that my analysis is inappropriate and/or not rigorous. Reviewer 2 says, "Some aspects of the suggested approach are presented mathematically, but unfortunately with several errors and imprecisions (e.g. unclear distinction between new definitions and consequences of previous definitions)." In its current form, this criticism is meaningless, as no actual errors or imprecisions have been identified. If specific instances, as above, can be identified where mathematical rigour is lacking, I would be glad to tighten these up.

5. Reviewer 2 also comments twice that they would like to see some traditional simulation work. I feel that this request misses the point of what I am proposing in this paper. Traditionally, simulation studies are used to compare the average performance of two or more competing methods against a variety of different criteria, e.g. bias of estimators for various sample sizes, behaviour of their MSEs, etc. However, such simulations always presuppose that the *sample data* are the same across all competing methods; i.e. it is only the *model* or, more broadly, the statistical technique that varies across conditions. The common dataset allows one to meaningfully compare, using one or more metrics, the performance of one technique against another.

   By definition, however, RVVMs generate *different sample data* than most traditional methods in applied practice. That is, the incorporation of response process

error via RVVMs generates non-deterministic sample data (i.e. nontrivial probabiliy measures), whereas traditional methods only produce sample measurements that are fixed real numbers (i.e. trivial, or point-mass, probability measures). Consequently, simulation studies are not directly meaningful. The classical approach ignores response process error, while RVVMs encode it. This is not a difference in model choice or statistical technique. This is a difference in raw sample data. Concerns like "the average performance of the methods over repeatedly simulated datasets" are not directly meaningful; "performance" as compared to what? The single datasets that I have simulated in my worked examples of Section 4 are sufficient to illustrate the differences between the RVVM approach to sample measurement and the classical approach. Additional simulated datasets would simply repeat the same lessons. It is not "average differences" that are important; it is *exact, sample-unit-specific* differences that matter here.
